# Supplementary material for: Modeling glioblastoma heterogeneity as a dynamic network of cell states
Source: Mol Syst Biol. 2021 Sep 16;17(9):e10105. doi: 10.15252/msb.202010105 (PMC8444284; doi:10.15252/msb.202010105)
Supplement: Supplementary file 6 — Source Data for Figure 5 [file MSB-17-e10105-s004.zip › Figure5A_sourcedata/GSEA_3071/nosigdb_stateII.GseaPreranked.1622795151171/RICHARDS_NEFTEL_CELL_2019_G1_S.html]

Details for gene set RICHARDS\_NEFTEL\_CELL\_2019\_G1\_S[GSEA]

|  || Dataset | state23071 |
| Phenotype | NoPhenotypeAvailable |
| Upregulated in class | na\_pos |
| GeneSet | RICHARDS\_NEFTEL\_CELL\_2019\_G1\_S |
| Enrichment Score (ES) | 0.8878454 |
| Normalized Enrichment Score (NES) | 3.7593803 |
| Nominal p-value | 0.0 |
| FDR q-value | 0.0 |
| FWER p-Value | 0.0 |
Table: GSEA Results Summary

  

Fig 1: Enrichment plot: RICHARDS\_NEFTEL\_CELL\_2019\_G1\_S      
 Profile of the Running ES Score & Positions of GeneSet Members on the Rank Ordered List

  

| PROBE | GENE SYMBOL | GENE\_TITLE | RANK IN GENE LIST | RANK METRIC SCORE | RUNNING ES | CORE ENRICHMENT || 1 | HIST1H4C |  |  | 2 | 1.902 | 0.0601 | Yes |
| 2 | RRM2 |  |  | 3 | 1.857 | 0.1191 | Yes |
| 3 | ZWINT |  |  | 16 | 1.510 | 0.1643 | Yes |
| 4 | FEN1 |  |  | 19 | 1.464 | 0.2104 | Yes |
| 5 | RAD51AP1 |  |  | 25 | 1.384 | 0.2532 | Yes |
| 6 | CENPK |  |  | 26 | 1.382 | 0.2972 | Yes |
| 7 | RRM1 |  |  | 37 | 1.310 | 0.3364 | Yes |
| 8 | GINS2 |  |  | 40 | 1.306 | 0.3775 | Yes |
| 9 | CDCA5 |  |  | 41 | 1.289 | 0.4185 | Yes |
| 10 | TK1 |  |  | 42 | 1.281 | 0.4593 | Yes |
| 11 | AURKB |  |  | 44 | 1.276 | 0.4997 | Yes |
| 12 | MCM7 |  |  | 45 | 1.273 | 0.5402 | Yes |
| 13 | PCNA |  |  | 58 | 1.214 | 0.5759 | Yes |
| 14 | CDC45 |  |  | 59 | 1.212 | 0.6145 | Yes |
| 15 | MELK |  |  | 69 | 1.145 | 0.6487 | Yes |
| 16 | CCNE2 |  |  | 76 | 1.121 | 0.6829 | Yes |
| 17 | MCM4 |  |  | 81 | 1.110 | 0.7173 | Yes |
| 18 | GMNN |  |  | 83 | 1.108 | 0.7523 | Yes |
| 19 | MCM3 |  |  | 105 | 1.035 | 0.7801 | Yes |
| 20 | DUT |  |  | 118 | 1.009 | 0.8093 | Yes |
| 21 | ASF1B |  |  | 120 | 1.007 | 0.8411 | Yes |
| 22 | CKS1B |  |  | 128 | 0.999 | 0.8712 | Yes |
| 23 | CENPM |  |  | 178 | 0.901 | 0.8878 | Yes |
| 24 | PKMYT1 |  |  | 322 | 0.736 | 0.8762 | No |
| 25 | TMEM106C |  |  | 377 | 0.694 | 0.8850 | No |
| 26 | MCM2 |  |  | 734 | 0.521 | 0.8142 | No |
| 27 | RNASEH2A |  |  | 1341 | 0.369 | 0.6771 | No |
Table: GSEA details [plain text format]

  

Fig 2: RICHARDS\_NEFTEL\_CELL\_2019\_G1\_S: Random ES distribution      
 Gene set null distribution of ES for **RICHARDS\_NEFTEL\_CELL\_2019\_G1\_S**
